# Targeting Viral Surface Proteins through Structure-Based Design

**DOI:** 10.3390/v13071320

**Published:** 2021-07-08

**Authors:** Yogesh B Narkhede, Karen J Gonzalez, Eva-Maria Strauch

**Affiliations:** 1 Department of Pharmaceutical and Biomedical Sciences, University of Georgia, Athens, GA 30602, USA; Yogesh.Narkhede@uga.edu; 2 Institute of Bioinformatics, University of Georgia, Athens, GA 30602, USA; Karen.Gonzalez@uga.edu

**Keywords:** vaccine, glycoproteins, structural vaccinology, rational design, computational protein design, respiratory viruses

## Abstract

The emergence of novel viral infections of zoonotic origin and mutations of existing human pathogenic viruses represent a serious concern for public health. It warrants the establishment of better interventions and protective therapies to combat the virus and prevent its spread. Surface glycoproteins catalyzing the fusion of viral particles and host cells have proven to be an excellent target for antivirals as well as vaccines. This review focuses on recent advances for computational structure-based design of antivirals and vaccines targeting viral fusion machinery to control seasonal and emerging respiratory viruses.

## 1. Introduction

Various spontaneous host adaptations of zoonotic viruses have led to highly infectious diseases in humans [1,2,3,4,5,6,7,8,9]. As a serious concern for public health, there is a demand for better monitoring and the need to develop therapeutic interventions and preventive care in form of vaccines. Respiratory viruses such as influenza and the recent coronaviruses have caused devastating pandemics with millions of fatalities globally [10,11]. While new influenza strains may derive from poultry or swine, such as the 2009 H1N1 pandemic [3,4], the most common source of human-transmissible and highly pathogenic beta coronaviruses is bats [12,13], often evolving first in disputed intermediate hosts, such as civets for severe acute respiratory syndrome coronavirus (SARS-CoV) [14], possibly pangolin for SARS-CoV-2 [15,16], and dromedary camels for Middle East respiratory syndrome (MERS) [17,18,19]). Studies on host adaptation in the context of positive selection have been controversial [20,21]. However, with the advent of next-generation sequencing, monitoring is advancing as more strain variations are collected [22], and detailed mutational maps are examined [23,24,25].

The most cost-effective and potentially long-term solution to fighting a viral disease is the development of vaccines. However, other means to tackle these infections include the use of small molecule antivirals and protein-based inhibitors, such as antibodies. With the emergence of the coronavirus disease of 2019 (COVID-19), it became apparent that adaptable platform technologies are needed for the rapid development and large-scale production of vaccines. The first two COVID-19 vaccines approved on an emergency basis by the FDA were based on an mRNA platform that had never before made it past clinical trials [26,27]. The efficacy of these mRNA-based vaccines was proven to be above 94% [28,29,30,31,32], and their manufacturing more efficient than, for example, recombinant proteins [33]. Given this development, we will likely see shifts in the vaccine development landscape. It is noteworthy that the SARS-CoV-2 mRNA-based vaccines encode a stabilized version of the fusion protein or spike protein (S protein) of the virus [34,35]. Owing to extensive structural studies of SARS-CoV [34] and MERS fusion proteins [36], two mutations, which were crucial to solving these proteins’ structure, also made possible the development of effective vaccines against SARS-CoV-2 [34,35]. As fusion proteins have been a major target to fight respiratory diseases, we will review the use of these proteins to develop antivirals and vaccines while focusing on structure-based design approaches.

## 2. Viral Fusion Proteins

Fusion glycoproteins decorate the surface of enveloped viruses and are essential for their cell entry. As critical players in the infection process and primary proteins on the viral surface, fusion proteins are excellent targets for developing antivirals, and they constitute the primary immunogens of different vaccine modalities [37,38,39,40,41,42,43]. To induce the fusion of viral and cellular membranes, fusion proteins refold from a highly strained and unstable prefusion conformation to a highly stable postfusion conformation that provides the free energy proposed to catalyze the fusion between host and viral membranes [44]. There are three different classes of fusion proteins—classes I, II, and III [44,45,46,47,48]. Despite sharing a general fusion mechanism, these proteins are structurally diverse and require different triggers to be activated. Class I fusion proteins are trimers characterized by a high content of α-helices, with a postfusion conformation displaying an α-helical coiled coil surrounded by three C-terminal helices (six-helix bundles) [45,49]. In contrast, class II fusion proteins are homo- or heterodimers in their prefusion state, and trimers in their postfusion state with a structural signature of β-sheets in both conformations [50]. Finally, class III fusion proteins are trimers in the prefusion and postfusion states and present a combination of α-helical and β-sheet structures [51]. In regard to activation mechanisms, class I and II fusion proteins require proteolytic processing either on themselves or on companion protein, whereas class III fusion proteins might not need processing to promote cell entry [52]. Class I fusion proteins are triggered by diverse factors that we will discuss in more in detail below [52]. Class II fusion proteins, once they are fusion competent, are mainly triggered by low pH, and class III fusion proteins are activated by low pH or interactions of a partner protein with a host cell receptor. Remarkably, while most viral fusion proteins undergo irreversible conformational changes upon activation, class III fusion proteins can achieve a thermodynamic equilibrium between the prefusion and postfusion states that allows the transition to be reversible [53,54].

**Figure 1:**
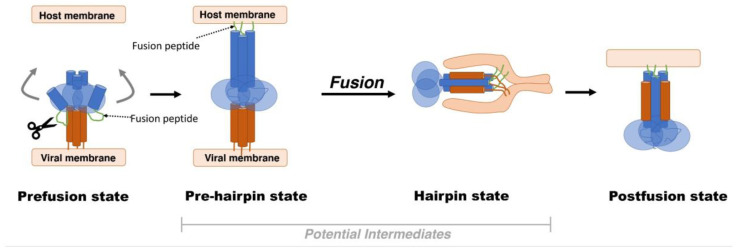
Model for membrane fusion; viral surface proteins undergo drastic conformational changes in order to bring the viral and host cell membrane close to each other. Upon its activation through proteolysis, the metastable prefusion state undergoes conformational changes in the fusion subunit that result in an intermediate state termed the “prehairpin” state [65,66,67,68]. At this point, the prehairpin structure can revert to its prior state in the absence of any membrane or irreversible transition to the postfusion state [48,69,70]. Finally, to prompt the fusion process, a short hydrophobic peptide or fusion peptide is released to connect with the target membrane. This interaction induces the formation of the six-helical postfusion state that brings the virus and host cell membranes in proximity and drives the membrane fusion.

In this review, we will focus on class I fusion proteins as they comprise the fusion machinery of numerous viral families such as the Orthomyxoviridae (e.g., influenza), Paramyxoviridae (e.g., respiratory syncytial virus-RSV), Coronaviridae (e.g., SARS-CoV-2), Retroviridae (e.g., HIV), and Filoviridae (e.g., Ebola) [44,45,48]. Class I fusion proteins, by far, are the most studied and are observed in numerous viral families of interest, including SARS-CoV-2. In the wake of the current pandemic, it is important to discuss class I fusion proteins as targets of clinical significance. As a generalized model, class I fusion proteins are initially synthesized as single chains precursors that become fusion competent by proteolytic maturation [52,55,56,57]. This state, known as the prefusion state, is characterized by a metastable structure that can easily be triggered to transition into a large, irreversible conformational change to the postfusion state while undergoing several intermediate states (Figure 1) [58,59,60,61]. Triggering factors include the switch to a low pH environment (e.g., influenza hemagglutinin (HA) protein) [44,48], interactions with coreceptors on the cell surface (e.g., the human immunodeficiency virus (HIV) Env protein interacting with the C–C chemokine receptor type 5 (CCR5)) [44], or interactions with cell surface receptors paired with localized protease cleavage (e.g., the SARS-CoV-2 S protein binding to the angiotensin-converting enzyme 2 (ACE2) receptor and is subsequentially cleaved by the transmembrane serine protease 2) [62,63,64].

The fusion process can be halted by stabilizing any of the conformations before the postfusion state is achieved. Alternatively, receptor binding can be inhibited to prevent attachment in the first place; both strategies are utilized by neutralizing antibodies. For HA, for instance, the receptor-binding site pocket is a conserved area in an otherwise variable region that is targeted by broadly neutralizing antibodies (bnAbs) [71,72,73], although their breadth of neutralization is limited due to high variability around this site. On the other hand, the HA stalk is rather conserved, which tends to confer anti-stem antibodies targeting a specific epitope close to the fusion loop—a very broad neutralizing potential.

The antivirals oseltamivir, zanamivir, and peramivir exhibit another inhibition mechanism targeting the neuraminidase protein and block the viral progeny release from infected cells. Though they do not directly involve the fusion proteins, they target a surface glycoprotein and interfere with the molecular mechanism. However, they are only effective shortly after infection [74].

## 3. Antivirals Targeting Viral Surface Proteins

Many small-molecule antiviral drugs reduce the viral load, disease symptoms, or mortality of a viral infection by directly targeting the replication of the virus in the host cells. This is exemplified by the current antiviral drugs against HIV (abacavir; Ziagen), herpes simplex virus (famciclovir; Famivir) [75], and hepatitis viruses (lamivudine; Epivir) [76]. However, only a few options are available for respiratory viruses, such as RSV, influenza, or coronaviruses.

RSV infections are treated often by supportive care (e.g., acetaminophen for fever and intravenous fluids for dehydration) or neutralizing antibodies, such as palivizumab [77]. However, antibody-based treatments can quickly become very expensive. There are four FDA-approved small molecule antivirals targeting influenza viruses. Among these, drugs such as oseltamivir phosphate (oral, Tamiflu^®^), zanamivir (inhaled, Relenza^®^), and peramivir (intravenous, Rapivab^®^) target the influenza neuraminidase protein and are active against influenza A and B viruses. However, these drugs are often only effective when given early after infection. Although small-molecule antivirals and their combinations have proven to be valuable, research and development of new small molecules against an emerging pandemic such as COVID-19 have been challenging [78,79,80]. In addition to a long development timeline (about a decade from bench to market), hurdles include potential off-site effects due to their limited specificity and often weak affinities (given the small surface area of small molecules) that result in adverse side effects or the need for frequent doses to achieve beneficial therapeutic endpoints. In contrast to developing new small molecules, drug repurposing is a promising alternative to treat viral diseases as already approved drugs can target other pathogenic viruses [80]. This was the case for remdesivir (Veklury^®^, Gilead Sciences, Foster City, CA, USA), which was originally developed for Ebolavirus yet currently holds an emergency FDA approval for cases of COVID-19 [81,82]. Likewise, the antiviral T-705 (favipiravir), which was initially approved for use against influenza in Japan [83], is now being assessed for protection against SARS-CoV-2 infections [84]. Both molecules are prodrugs that are phosphorylated in vivo to their triphosphate forms, which target the viral RNA-dependent RNA polymerase (RdRp). The bioactive triphosphates compete with purine nucleosides to incorporate into viral RNA and interfere in its elongation and viral proliferation.

Protein-based therapeutics represent another option for treating viral infections. The large protein–protein or protein–glycan interfaces enable higher affinity and specificity than small-molecule drugs. Despite their high manufacturing cost and possible low stability, monoclonal antibodies constitute the largest set of protein-based therapeutics against a wide variety of diseases (bacterial and viral infections, cancer, arthritis, etc.). Antibody-based therapeutics has also been a life-saving approach during the current COVID-19 pandemic. Particularly, the Regeneron cocktail (REGN-COV^TM^, Regeneron Pharmaceuticals, New York, NY, USA) (casirivimab with imdevimab) [85], and Eli Lilly’s treatment option (bamlanivimab with etesevimab) [86,87], which target different sites of the receptor-binding domain of the S protein, have achieved FDA emergency use approval to protect against SARS-CoV-2. These antibodies were developed at remarkable speed, given the onset of the pandemic in February 2020, their discovery in May 2020, and the clinical trials and FDA approval in November 2020 and February 2021, respectively [88].

**Figure 2:**
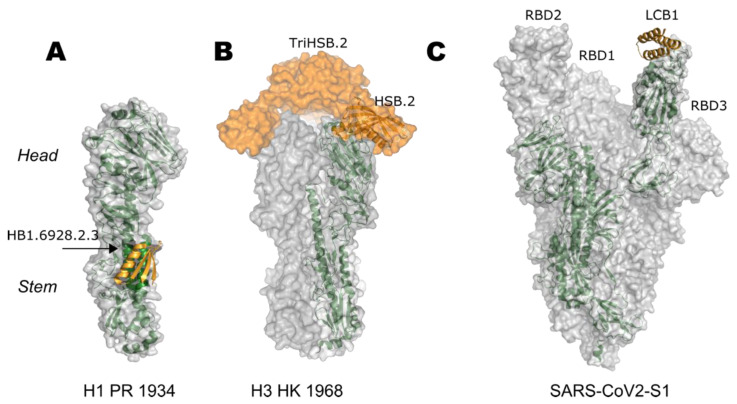
Designed small protein inhibitors targeting fusion proteins. All proteins have been depicted as transparent surfaces with monomeric units highlighted in forest green. Protein inhibitors are colored orange (**A**) HSB1.6928.2.3 targeting the stem region of H1 HA (PDB 5VLI); (**B**) TriHSB.2A targeting the receptor-binding site on the head region of H3 HA 1968 strain (PDB 5KUY and model); (**C**) LCB1 bound to the open conformation of the receptor-binding domain of prefusion stabilized ectodomain trimer of SARS-CoV-2 spike protein (PDB 7JZL).

An alternative route to large antibody molecules is the use of small protein-based inhibitors. As seen in naturally occurring inhibitors where small proteins bind to larger proteins to occlude their activity (e.g., cystatin in trypsin [89]), it is possible to develop small, highly stable, and simple-to-produce proteins that can block functional sites in a target biomolecule. Recent advances have demonstrated that computational design can generate protein–protein inhibitors with extraordinary potency. As an example, inhibitors designed de novo against different regions of the influenza HA protein have successfully hindered the protein’s ability to mediate the fusion process [90,91,92] or bind to human cell receptors [93]. Both types of inhibitors have neutralized the influenza virus and protected mice prophylactically and therapeutically (Figure 2). Most recently, small protein inhibitors were developed to block the receptor-binding site of the SARS-CoV-2 S protein, resulting in highly stable picomolar inhibitors (Figure 2) [94]. These optimized molecules neutralized the virus in Golden Syrian hamsters and had 3 days prophylactic and 21 days therapeutic effects [95]. Given its small molecular weight of less than 5 kDa, compared to the average 150 kDa of a monoclonal antibody, much less material needs to be produced in order to have the same efficacy and thereby presents an excellent opportunity to provide for a larger number of people. The strong binding of these designed small proteins is one of the main advantages of using computational design over traditional methods. In contrast to monoclonal antibodies, which are usually identified out of a pool, computational protein design confers total control over the regions to be targeted by the inhibitor and allows the optimization of desired interactions. Lastly, small protein inhibitors are a promising approach to fight viral infections as their high stability could eliminate storage and transportation barriers that usually frustrate the delivery of treatments to remote areas.

## 4. Rational Structure-Based Vaccine Design and Development

While both the prefusion and postfusion states of the fusion proteins have been studied as vaccine candidates, the prefusion conformation has been shown to elicit more potent antibodies [96,97,98,99,100]. Notably, stabilized prefusion proteins have been demonstrated to improve the immunogenicity of diverse vaccine formulations such as protein-based vaccines [36,39,41,101], virus-like particles [102], gene-based vectors [42], and nucleic acid-based vaccines [29,30]. Stable prefusion proteins have also been essential in the identification of potent neutralizing antibodies that can serve as prophylactic and/or therapeutic agents [97,103,104].

**Figure 3:**
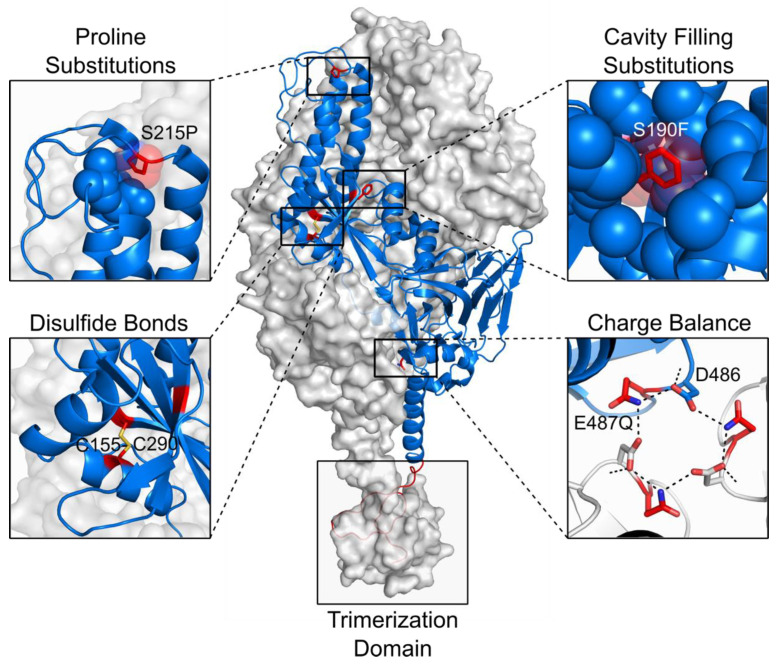
Strategies to stabilize the prefusion conformation of class I fusion proteins. The protein shown corresponds to the trimeric RSV F protein (PDB 4MMV and 5C6B) with two protomers as grey molecular surfaces and one protomer as a blue ribbon. Stabilizing substitutions (S215P, S190F, S155C, S290C, Q487, and a foldon domain) are presented in red, and hydrogen bonds are depicted as black dotted lines. Each panel contains an example of the main stabilization strategies of the prefusion conformation.

The successful application of structure-based design approaches to stabilize the prefusion conformation of the RSV fusion protein (F protein), has provided the basis for generalized strategies to stabilize class I fusion proteins [41]. This pioneering work, which led to the clinical candidate DS-Cav1, was aimed to design prefusion RSV F variants with a stabilized antigenic site Ø. The strategy involved designing a disulfide bond that prevents the postfusion state, introducing two cavity-filling substitutions that increase favorable interactions and the structural order of the protein, and appending a C-terminal T4-phage fibritin trimerization domain (“foldon”) that preserves the protein’s trimeric structure (Figure 3) [41]. The effectiveness of this approach was later supported by the massive stabilization of the prefusion F protein of the four different types of human parainfluenza viruses [43].

A closer analysis of the refolding mechanism of RSV F further identified that proline substitutions at hinge loops can halt the transition from the prefusion to the postfusion conformation (Figure 3). By disrupting the extension of the central helices that form the postfusion helical bundle, proline mutations have had a remarkable impact on the prefusion stabilization of fusion proteins of RSV [105], human metapneumovirus (hMPV) [106], MERS, SARS-CoV, and SARS-CoV-2 [35,36,107,108]. As mentioned above, the rapid study of the S-protein of the novel SARS-CoV-2 was the result of increased stability of the protein’s prefusion state by introducing two proline mutations [34]. The expression levels and stability of this prefusion SARS-CoV-2 S protein were additionally improved by four more proline substitutions that rigidified flexible loops or stabilized the N termini of helices in the fusion peptide and regions around it [108]. Notably, although the SARS-CoV-2 S protein was also stabilized by cavity filling substitutions and the introduction of salt bridges and disulfide bonds, the most substantial increase in expression and stability has been observed in proline-containing variants [107,108].

The neutralization of charge imbalances, particularly at the interface between protomers, as well as the replacement of the peptide between maturation cleavage sites by a short linker, represent additional strategies that have highly contributed to increasing the expression levels of different fusion proteins (Figure 3) [105,109,110,111]. Finally, a strategy to promote the trimerization of the soluble protein without appending a foldon domain has been successfully designed by introducing a ‘‘cysteine zipper’’ at the C terminus of the RSV F protein (Figure 3). The removal of nonpathogenic motifs such as the foldon is of critical importance in vaccine development to reduce potential off-target reactivity [112].

## 5. Epitope-Focusing through De Novo Scaffolding

**Figure 4:**
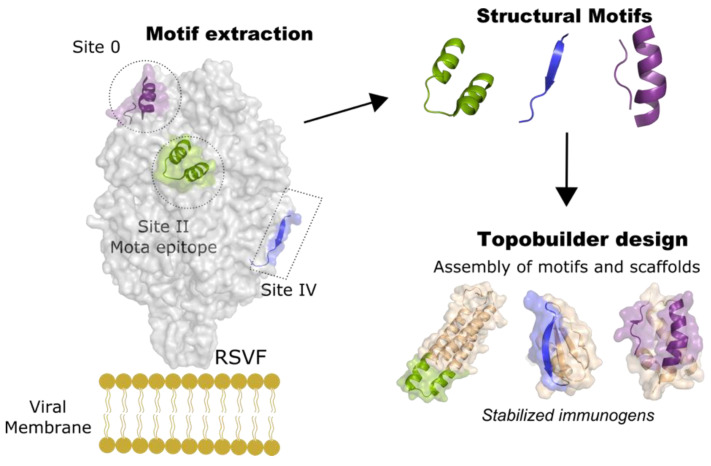
Strategy for de novo design of a trivalent epitope-focused vaccine. The neutralizing sites 0, II, and IV of RSV F were stabilized by de novo designed scaffolds using Topobuilder [121]. Topobuilder aided the construction of specific topologies that stabilize the antigenic motifs of the RSV F protein. Subsequent design and folding simulations yield stable immunogens that were used to generate a tri-scaffold vaccine. The combination of three scaffolds induced specific neutralizing antibodies against RSV F in nonhuman primates.

Epitope-focused vaccine design is a promising approach for developing immunogens that direct the immune response toward specific structural epitopes [113]. Driven by the need to develop vaccines against highly antigenically variable viruses, such as HIV, this strategy has been actively used to present specific conserved epitopes out of context. The initial epitope-focused method involved transplanting single epitopes that bind to broadly neutralizing antibodies onto new, small, and optimized scaffolding proteins. This approach, which originally used only side-chain grafting and later grafting of entire regions (continuous and discontinuous) of the viral surface proteins of HIV and RSV [114,115,116,117,118], promoted structure-specific but not necessarily neutralizing antibodies. Owing to the recent progress in de novo protein design, the method was extended to build new proteins “around” the epitopes [113]. With this variation, the design of scaffold proteins containing the helix–turn–helix epitope of the RSV F protein (PDB 3IXT), which is recognized by the neutralizing antibody motavizumab (mota) [119,120], produced immunogens with better thermal stability and biophysical characteristics and also protected rhesus macaques against RSV [113]. Further improvement comprised the design of template-free scaffolds for structurally complex and discontinuous neutralizing epitopes, using the computational protocol named Topobuilder [121]. As a proof of concept, the prefusion conformation of the RSV F protein (PDB 4JHW) [103] was used to design immunomimetics for the asymmetric and intermittent neutralizing epitopes sites 0 [103] and IV [122], as well as for the continuous mota epitope (site II) (Figure 4). Remarkably, when boosting with the epitope scaffolds after priming with the RSV F protein, the designed immunogens elicited higher antibody titers against sites 0, II, and IV than boosting with the wild-type F protein. Furthermore, nonhuman primates vaccinated with Trivax1 (an equimolar combination of all 3 epitope presenting proteins) produced robust levels of cross-reactive serum titers against RSV F, with the three sites being targeted equally [121]. These findings are evidence that Topobuilder provides a promising epitope-focused vaccine approach, with an exceptional degree of control over antibody specificities across naïve and primed antibody expression profiles.

## 6. Self-Assembling, Designed Nanoparticles for Geometric-Defined Oligomeric Display of Antigens

Surface glycoproteins can be found at different densities on the viral envelope depending on the virus. While HIV has a sparse decoration of the envelope protein on its surface [123], influenza displays a tight network of HA and neuraminidase molecules [124]. Since the immune system is constantly exposed to this diverse array of multimeric glycoproteins, strategies including the oligomeric display of surface antigens are valuable for vaccine development. As a pioneering work, Kanekiyo et al. demonstrated that robust immune responses could be elicited when displaying HA proteins as a fusion to the self-assembling, protein-based ferritin nanoparticle [125].

**Figure 5:**
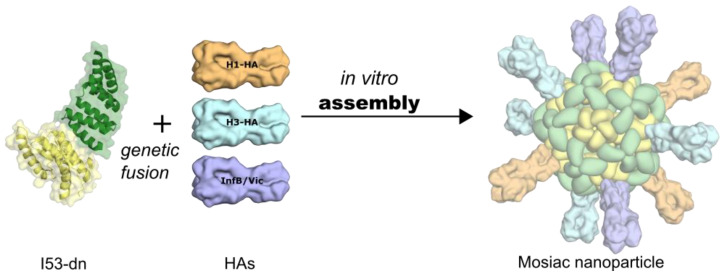
De novo design of self-assembling mosaic nanoparticles displaying various HA antigens.

Using the Rosetta software suite, King et al. also developed an innovative approach for designing cage-like nanomaterials by applying a combination of symmetric docking and optimization of protein–protein interfaces [126,127]. This process was first illustrated for a dual tetrahedron (T33) in which four copies, each with two distinct trimeric building blocks were placed at opposite ends of the threefold symmetric tetrahedral axes. Subsequentially, different assemblies, including two-component viral capsid-like shapes, have been generated and tested for the display of viral proteins as oligomeric immunogens [128]. In this regard, Ueda et al. designed protein-based nanoparticles that enabled the multivalent presentation of homo-oligomeric class I fusion proteins such as HIV-I Env, influenza HA, and the RSV F protein in their prefusion conformations [129]. The design of these multivalent de novo designed protein nanoparticles showed that proximal geometrical placement of termini of the antigens and the nanoparticle subunits would enable multivalent presentation, better stability, and unprecedented control over the antigen presentation [129]. Indeed, increased antibody titers were observed for the self-assembling de novo designed nanoparticle DS-Cav1-I53-50 displaying multivalent (20) copies of DS-Cav1 [39]: DS-Cav1-I53-50 induced threefold higher antigen-specific and ninefold higher neutralizing antibody titer in mice when compared to the recombinant DS-Cav1, with similar profiles in nonhuman primates. Furthermore, it was demonstrated that a two-component, icosahedral nanoparticle (I53-50) displaying the receptor-binding domain (RBD) of the SARS-CoV-2 S glycoprotein also increased the neutralizing antibody response in mice and nonhuman primates [130]. Most recently, the quadrivalent display of different HA variants of four influenza vaccine strains (of years 2017, 2018, and 2019) on the same nanoparticle (qsMosaic-I53_dn5) presented the first hetero-oligomeric particle (Figure 5) that results in broader protection against multiple flu strains than the currently administered vaccines, in mice, ferrets, and nonhuman primates [131]. The assumption is that the robust antibody response for each of the protein nanoparticles stems is partially due to a dense array of displayed antigens that lead to efficient B-cell receptor crosslinking [39] though precise display geometry plays a central role as well.

## 7. Targeting Sequence Shape Shifters: Influenza

As influenza viruses use antigenic variability to escape from the immune response, one of the major challenges in developing influenza vaccines is in conferring effective protection against the diverse antigenic forms of the virus [132]. Since current influenza vaccines are only effective when the circulating strain is antigenically similar to the vaccine strains, the design of broadly reactive or universal influenza vaccines is still an unmet need [133].

During naturally occurring infections or after the influenza vaccination, neutralizing antibodies are mostly produced against the viral HA protein [134,135]. This HA protein is structurally composed of two domains known as the head domain, which mediates the attachment of the virus to the host cell receptor, and the stem domain, which promotes the fusion between the viral and the cell membranes [136]. While the head domain denotes the immunodominant region of HA proteins and comprises most of their antigenic variability, the stem subunit is a more conserved subdominant domain [137]. Antigen design strategies focusing on HA proteins have shown promising progress toward the development of broadly reactive influenza vaccines. These design advances are divided into two main avenues: head-based and stem-based approaches.

### 7.1. Head-Based Vaccine Design

**Figure 6:**
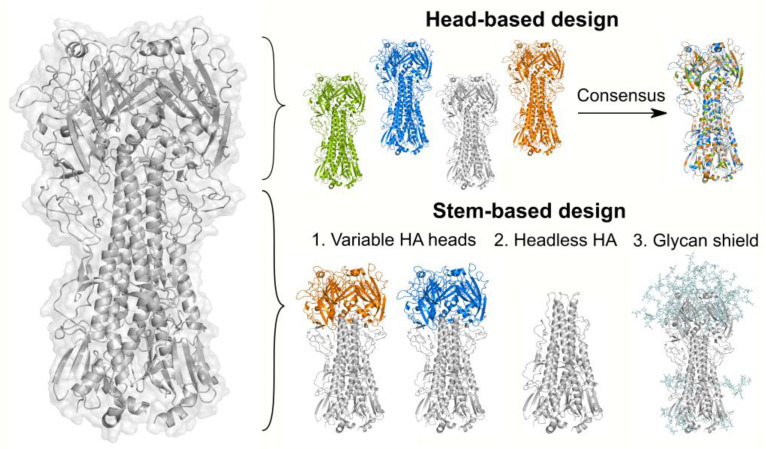
Strategies to design broadly reactive HA-based influenza vaccines. The top panel depicts the COBRA design technology. The COBRA strategy uses diverse HA sequences and multiple rounds of consensus sequence calculations to generate a unique immunogen that can elicit head-targeting antibodies. The bottom panel shows the main protein-design approaches to redirect the immune response towards the conserved HA stem domain. These strategies include (1) chimeric HA constructs consisting of a conserved HA stem domain (gray) and distinct HA heads from viruses absent in humans (blue and orange regions); (2) headless HA proteins designed by removing the HA head domain and introducing stabilizing substitutions at the stem; (3) modifications of HA glycosylation sites to hide immunodominant epitopes at the HA head domain (e.g., hyperglycosylation of the head domain). In this panel, the HA protein is shown in gray, while glycans are shown in light blue. The glycans displayed are an artificial representation of this strategy and were drawn using GlyProt [142]. All figures were produced using PyMol [143] and the PDB 4m4y [144].

As the head domain contains the receptor-binding site, most neutralizing antibodies target this region to block the virus from binding to the host cell receptors [138]. Given this immunologic pressure, epitopes at the globular head are continuously mutating to avoid antibody recognition. Although targeting the head represents a great challenge due to its antigenic variability, one protein design approach known as “computationally optimized broadly reactive antigens (COBRA)” has been demonstrated to overcome the head’s variability and produce immunogens eliciting broad immune responses [139,140,141]. COBRA technology consists of a multilayer consensus design of HA sequences. The HA sequences are initially grouped by antigenic eras or phylogenetic subclades, followed by different rounds of consensus sequence calculations (Figure 6). The final consensus sequences designed with this method have successfully shown broad reactivity in H5N1, H1N1, and H3N2 isolates [139,140,141].

### 7.2. Stem-Based Vaccine Design

A strategy to overcome antigenic variability is to direct the immune response towards the conserved regions of the target immunogen. In HA proteins, the stem is highly conserved across different influenza subtypes, and antibodies targeting this region have proven to be broadly reactive [145,146,147,148,149]. However, due to the immunodominance of the HA globular head, stem-directed immunity is minimally induced by vaccination or exposure to influenza [150]. Different studies have revealed that stem-reactive antibodies can be boosted when the dominant epitopes are inaccessible, or when there is continuous exposure to antigenically divergent HA heads [150,151,152,153,154]. Based on these observations, three main strategies have been successfully developed to elicit stem antibodies (Figure 6).

#### 7.2.1. Immunization with Antigenically Variable Globular Heads

Sequential immunizations with synthetic chimeric HAs (cHAs) containing a conserved stem domain but holding divergent globular heads have been demonstrated to boost stem-specific antibodies [155,156,157,158]. These chimeric proteins are engineered by combining the stem subunit from one influenza subtype with an irrelevant head from viruses absent in humans. In this approach, B cells recognizing both head and stem epitopes are generated during the primary immunization. However, upon subsequent immunizations, the preexisting memory B cells are recalled for conserved antigens. Since the immunodominant head epitopes are antigenically distinct in each immunization, only stem-specific antibodies are boosted [135]. This chimeric-HA immunization strategy is currently undergoing clinical trials [159]. One variation of this approach has also shown promising results in the development of universal influenza vaccines. In this modification, only the major antigenic sites are replaced by diverse HA sequences rather than the entire globular head. The resulting mosaic proteins are intended to boost the antibody response for stem antigens and for conserved epitopes at the head [160,161,162].

#### 7.2.2. Removal of the Globular Head

Analogous to the previous strategy, HA variants lacking the globular head or “headless” proteins have been shown to efficiently elicit anti-stem antibodies [146,163,164,165,166,167,168,169,170,171,172]. As the removal of the head destabilizes the stem subunit, the incorporation of a trimerization domain or the fusion of the stem antigen to a self-assembling ferritin nanoparticle has been necessary to ensure the structural integrity of these immunogens. Both stabilized stem immunogens have been proven to elicit broad immunity and are promising candidates as broadly protective vaccines. The ferritin nanoparticles are currently being evaluated in clinical trials [173].

#### 7.2.3. Glycan-Masking of Immunodominant Epitopes

The third approach to redirect the immune response to the HA stem consists of concealing immunodominant epitopes at the globular head by introducing new N-glycosylation sites. The glycosylation of these additional spots obstructs the access of antibodies to the main head epitopes and facilitates the recognition of other immune, subdominant regions [174,175,176,177]. The capacity to drive the immune response toward specific regions of the HA protein has made this approach an attractive method of identifying novel antigenic epitopes at both the head and the stem domains [176]. In addition to masking immunodominant regions, modifications in the HA glycosylation pattern have also been used to increase the immunogenicity of conserved epitopes. In this regard, the removal of certain glycans around stem epitopes has been found to induce a more potent immune response against homologous, heterologous, and heterosubtypic influenza viruses [178].

## 8. Conclusions

The past decade has witnessed rapid strides in the development of methods for the computer-aided design of immunogens and protein-based inhibitors that mainly target viral fusion proteins. These successful approaches imply remarkable preclinical and recent clinical progress toward potent vaccine candidates broadly effective against respiratory viruses. We believe they are promising solutions for the development of preventative and therapeutic measures to tackle current and future infectious diseases.
